# Cell-Penetrating Peptide-Mediated Delivery of TALEN Proteins via Bioconjugation for Genome Engineering

**DOI:** 10.1371/journal.pone.0085755

**Published:** 2014-01-20

**Authors:** Jia Liu, Thomas Gaj, James T. Patterson, Shannon J. Sirk, Carlos F. Barbas III

**Affiliations:** The Departments of Chemistry and Cell and Molecular Biology, The Skaggs Institute for Chemical Biology, The Scripps Research Institute, La Jolla, California, United States of America; Université Paris-Diderot, France

## Abstract

Transcription activator-like (TAL) effector nucleases (TALENs) have enabled the introduction of targeted genetic alterations into a broad range of cell lines and organisms. These customizable nucleases are comprised of programmable sequence-specific DNA-binding modules derived from TAL effector proteins fused to the non-specific FokI cleavage domain. Delivery of these nucleases into cells has proven challenging as the large size and highly repetitive nature of the TAL effector DNA-binding domain precludes their incorporation into many types of viral vectors. Furthermore, viral and non-viral gene delivery methods carry the risk of insertional mutagenesis and have been shown to increase the off-target activity of site-specific nucleases. We previously demonstrated that direct delivery of zinc-finger nuclease proteins enables highly efficient gene knockout in a variety of mammalian cell types with reduced off-target effects. Here we show that conjugation of cell-penetrating poly-Arg peptides to a surface-exposed Cys residue present on each TAL effector repeat imparted cell-penetrating activity to purified TALEN proteins. These modifications are reversible under reducing conditions and enabled TALEN-mediated gene knockout of the human *CCR5* and *BMPR1A* genes at rates comparable to those achieved with transient transfection of TALEN expression vectors. These findings demonstrate that direct protein delivery, facilitated by conjugation of chemical functionalities onto the TALEN protein surface, is a promising alternative to current non-viral and viral-based methods for TALEN delivery into mammalian cells.

## Introduction

Zinc-finger nucleases (ZFNs), transcription activator-like (TAL) effector nucleases (TALENs) and CRISPR/Cas9-based systems are valuable reagents for inducing targeted genetic alterations within complex genomes [1], [2]. These nucleases generate DNA double-strand breaks (DSBs) that can be repaired by error-prone non-homologous end joining (NHEJ) or homology-directed repair (HDR) [3]. These strategies have enabled genome editing in diverse human cell types, including primary T lymphocytes [4], [5], embryonic and induced pluripotent stem cells [6]–[8] and hematopoietic progenitor/stem cells [9], [10], as well as in a broad range of organisms, including *Drosophila* [11], *C. elegans* [12], [13], rats [14], [15], mice [16], zebrafish [17]–[20], and livestock [21]. These technologies have also advanced the promise of gene therapy, as site-specific nucleases have the potential to correct the underlying causes of numerous genetic diseases in humans. Most notably, ZFN-induced gene knockout of the human *C-C chemokine receptor type 5* (*CCR5*) gene has resulted in HIV-1 resistance in mouse models [4], [9] and is currently being evaluated in human clinical trials (NCT01252641, NCT00842634 and NCT01044654).

Although targeted nucleases provide researchers with a means for rapid and facile introduction of custom modifications at virtually any genomic locus, this technology remains limited by difficulties in delivery of the nucleases into cells. TALEN gene delivery, in particular, has proven more challenging than ZFN delivery due to the large size and highly repetitive nature of the TAL effector DNA-binding domain. A typical TAL effector consists of a series of 33- to 35-amino acid repeats that each recognizes a single base pair (bp) through two adjacent amino acid residues, termed the repeat variable di-residue (RVD) [22], [23]. Although this modularity allows creation of custom domains capable of recognizing virtually any DNA sequence [24], the repetitive structure of these units can result in rearrangements within TALEN genes when delivered into cells by lentiviral vectors [25]. Furthermore, the large size of the TALENs has thus far prevented their delivery into cells by space-constrained delivery vehicles such as adeno-associated virus [26], [27]. Transfection of TALEN-encoding plasmid DNA or mRNA offers an alternative to viral-based methods, but these approaches are restricted to certain cell types and can be highly toxic [28].

We previously showed that direct delivery of ZFN proteins yields highly efficient gene knockout in a variety of mammalian cell types [29]. Direct delivery of site-specific nuclease proteins offers several distinct advantages over methods that rely on expression from a viral vector or plasmid, including: (*i*) no risk of insertional mutagenesis, (*ii*) fewer off-target cleavage events due to reduced nuclease exposure time within the cell, (*iii*) reduced toxicity, and (*iv*) fewer regulatory concerns for genetic therapies and genetically modified foods and plants. The cell-penetrating properties of ZFN proteins, however, lie within the Cys_2_-His_2_ zinc-finger domain and are therefore absent from the TAL effector DNA-binding domain. Thus, in order for this technique to be applicable to TALENs, cell-penetrating activity must be artificially introduced into the TAL effector protein. Current approaches for imparting cell-penetrating activity onto site-specific nucleases rely on conjugation or fusion to functional domains, such as transferrin for receptor-mediated delivery [30] or protein transduction domains for direct membrane permeation [31], [32]. Here we explore the feasibility of reversibly conjugating cell-penetrating peptides to the surface of purified TALEN proteins. We demonstrate that TALEN proteins labeled with cell-penetrating poly-Arg peptides entered cells and efficiently induced gene knockout in transformed human cell lines with no overt toxicity. These results demonstrate that conjugation of cell-penetrating functionalities onto TALEN proteins is a promising alternative to current methods for delivering TALENs into mammalian cells.

## Materials and Methods

### Plasmid Construction

TALENs targeting the human *CCR5* gene [33] were kindly provided by Transposagen Biopharmaceuticals, and TALENs targeting the human *BMPR1A* gene [34] were obtained from Addgene (ID: TAL2260 and TAL2261). To construct bacterial TALEN expression vectors, the Sharkey cleavage domain was cloned into the pET-28 (+) expression vector (Novagen) as described [29]. TAL effector coding sequences were removed from mammalian expression vectors by digestion with NheI and BamHI and were ligated into the same restriction sites of the Sharkey-containing pET-28 expression vector to generate pET.TALEN.CCR5.L/R.SK and pET.TALEN.BMPR1A.L/R.SK. Each TALEN contained an N-terminal poly-His tag. Correct construction of each TALEN expression cassette was verified by sequence analysis (Table S1). Abbreviations are as follows: L, left TALEN; R, right TALEN; SK, Sharkey FokI cleavage domain.

### TALEN Expression and Purification

Chemically competent *Escherichia coli* BL21 (DE3) (Stratagene) were transformed with pET.TALEN.CCR5.L/R.SK and pET.TALEN.BMPR1A.L/R.SK. A single colony was added to 10 ml of LB medium in the presence of 50 µg/ml kanamycin, 200 mM NaCl, and 0.2% glucose. Bacteria were grown overnight at 37°C with shaking. The following day, 700 ml of LB medium supplemented with 50 µg/ml kanamycin, 200 mM NaCl, and 0.2% glucose was inoculated with 10 ml of the overnight culture and incubated at 37°C with shaking to an OD_600_ of 0.5, then incubated at room temperature with shaking to an OD_600_ of 0.8. TALEN synthesis was induced with 0.1 mM isopropyl β-D-1-thiogalactopyranoside (IPTG). After 4 hr, cells were harvested by centrifugation at 5,000 RCF for 10 min at 4°C, and the pellet was resuspended in 20 ml lysis buffer (50 mM sodium phosphate, pH 8.0, 500 mM NaCl, 1 mM MgCl_2_, 1× Complete Protease Inhibitor Cocktail (Roche), 1 mM β-mercaptoethanol, 10% glycerol). Cells were lysed by sonication, and the soluble fraction was centrifuged at 25,000 RCF for 30 min at 4°C. Lysate supernatant was filtered through a 0.45 µM low-protein binding filter (EMD Millipore). TALEN proteins were purified using Ni-NTA agarose resin (QIAGEN) and eluted with lysis buffer. All proteins were subsequently concentrated using an Amicon Ultra-15 Centrifugal Filter Unit (EMD Millipore) and then centrifuged at 12,000 RCF for 5 min at 4°C to remove precipitates. Glycerol was added to the TALEN protein solution to a final concentration of 20% (v/v). Protein samples were filtered through a 0.22-µm low-protein binding filter (EMD Millipore), aliquoted, and stored at −80°C. Protein purities and concentrations were assessed by SDS-PAGE. The protein yields after purification were between 2.0 and 5.0 mg/l.

### Peptide Conjugation

Purified left and right TALEN proteins (75 µl; 3.3 µM in 100 mM sodium phosphate with 1× Complete Protease Inhibitor Cocktail, pH 5.5) and 50 µM Cys (Npys)-(D-Arg)_9_ peptide (AnaSpec or Abgent) were combined and allowed to react at room temperature for at least 1 hr with no mixing. The pH was then neutralized with ∼0.1 volumes of 1 M sodium hydroxide. The reaction solution was then mixed with 175 µl serum-free Dulbecco’s modified Eagle’s medium (DMEM; Life Technologies) and centrifuged at 10,000 RCF for 5 min at 4°C to remove precipitated protein. Conjugated TALENs were directly applied to cells.

### *In vitro* Cleavage Assays

Cleavage assays were performed as described [35] with the following exceptions: The *CCR5* and *BMPR1A* TALEN target sequences were cloned into pUC19. Cleavage reactions contained 100 ng linearized DNA substrate, 50 mM potassium acetate, 20 mM Tris-acetate, 10 mM magnesium acetate, 1 mM DTT at pH 7.9 and indicated concentration of TALEN proteins. Cleavage reactions were performed at room temperature for 1 hr.

### TALEN Protein Treatments

HeLa and human embryonic kidney (HEK) 293 cells (American Type Culture Collection) were maintained in DMEM containing 10% (v/v) fetal bovine serum (FBS) and 1% Antibiotic-Antimycotic (Anti-Anti; Life Technologies). Cells (1×10^5^ cells per well) were seeded onto 24-well plates and established in a humidified 5% CO_2_ atmosphere at 37°C. After 24 hr, cells were washed with serum-free medium (SFM) and treated with TALEN proteins for 2 hr at 37°C. After treatment, cells were washed with SFM and maintained at 30°C with serum-containing medium for 24 hr, followed by incubation at 37°C for 24 hr. For transient expression experiments, cells were transfected with 200 ng of each TALEN expression vector 24 hr after seeding using Lipofectamine 2000 (Life Technologies) according to the manufacturer’s instructions. Cells were harvested 72 hr after transfection.

### Surveyor Nuclease Assay and Sequence Analysis

Genomic DNA was isolated using QuickExtract Extraction Solution (Epicentre), and the frequency of endogenous gene disruption was evaluated using the Surveyor nuclease assay (Transgenomics) as described [36]. The *CCR5* and *BMPR1A* genes were amplified by nested PCR using the Expand High Fidelity Taq System (Roche) and cloned into the plasmid pUC19 with the restriction sites EcoRI and BamH1. Sequence analysis was performed on individual cloned transformants as described [29]. Primer sequences are provided in Table S2.

### Cellular Proliferation Assays

HeLa and HEK293 cells were seeded onto 96-well plates at 1×10^5^ cells per well. At 24 hr after seeding, cells were treated with conjugated TALEN proteins as described above. Cell viability was measured using the Cell Proliferation Kit II (XTT; Roche Applied Science) according to the manufacturer’s instructions.

## Results

### TALEN Proteins Genetically Fused to Penetratin, Hph-1 and Transportan do not Enter Cells

As a first step toward generating cell-penetrating TALEN proteins, we fused the TAT [37], penetratin (Pnt) [38], Hph-1 [39], and transportan (Tp) [40] cell-penetrating peptides (CPPs) to the N-termini of TALENs designed to target the human *CCR5* gene [33]. As in previous work with ZFN proteins [29], we were unable to express or purify TAT-TALEN fusion proteins in yields high enough for analysis in cell culture, presumably due to low solubility. In contrast, TALEN proteins fused to the Pnt, Hph-1, or Tp transduction domains could be expressed and purified with modest-to-high yields (Fig. S1A). With the exception of the Tp-TALEN protein, each CPP-TALEN fusion protein demonstrated robust cleavage activities *in vitro* (Fig. S1B). To assess cell permeability, we treated HeLa cells with increasing amounts of each CPP-TALEN protein and evaluated endogenous *CCR5* gene modification using the Surveyor nuclease assay [36]. Under these conditions, none of the CPP-TALENs induced detectable levels of mutagenesis at the *CCR5* locus (Fig. S1C). When transiently transfected into HeLa cells using a CPP-TALEN expression vector, efficient gene disruption was observed (Fig. S1D). In order to determine whether CPP-TALEN proteins were capable of crossing cell membranes, we used Western blot to examine the lysate of HeLa cells treated with 2 µM of each CPP-TALEN protein. No TALENs was detected in any sample, indicating that cells do not take up CPP-TALENs from the surrounding medium (Fig. S1E). Taken together, these findings suggested that although the presence of the Pnt, Hph-1, or Tp CPPs did not negatively affect TALEN activity, these peptides were unable to confer cell-penetrating activity to TALEN proteins. While a recent study indicated that TAT-TALEN proteins are cell-permeable [31], the low yields obtained in our studies precluded their further development. We therefore sought to develop a more robust system for producing cell-permeable TALEN proteins.

### TALEN Proteins can be Reversibly Conjugated to Cell-penetrating Poly-Arg Peptides

**Figure 1:**
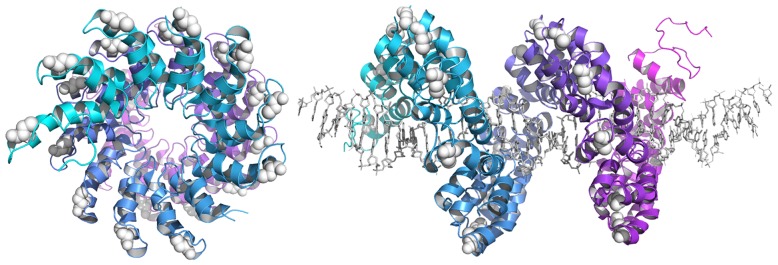
TAL effector structure. (Left) Front view of the PthXo1 DNA-binding domain in the absence of target DNA and (right) side view in the presence of target DNA. Surface-exposed Cys residues depicted as white spheres. TAL effector repeats are colored cyan and purple. DNA is shown as grey sticks. PDB ID: 3UGM [41].

**Figure 2:**
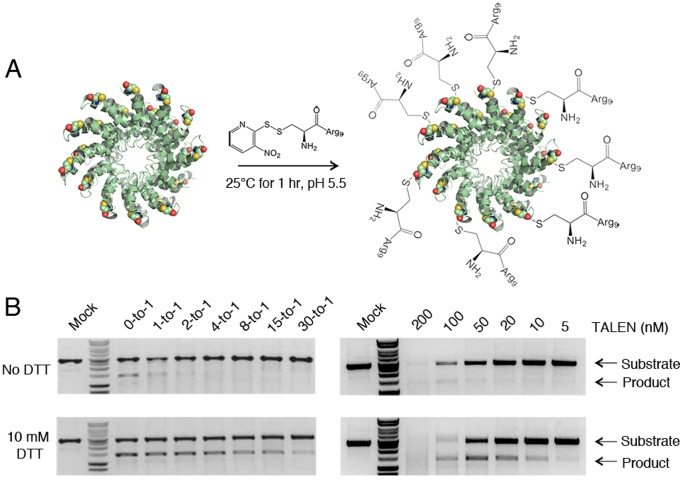
TALEN conjugation is reversible and R9 must be removed for TALEN cleavage activity. (A) Purified TALEN proteins are incubated with Cys-nitropyridyl (Npys) Arg_9_ cell-penetrating peptide (R9-CPP) for 1 hr at room temperature. (B) *In vitro* cleavage analysis of TALEN proteins conjugated at (left) various peptide-to-protein ratios and (right) various protein concentrations at a 30-to-1 peptide-to-protein ratio in the (top) absence or (bottom) presence of 10 mM DTT.

The crystal structures of the PthXo1 [41], dHax3 [42], and AvrBs3 [43] proteins revealed that each TAL effector repeat contains a single solvent-exposed Cys residue (Fig. 1). We hypothesized that conjugation onto these residues with CPPs would impart a degree of positive charge that would enable cell penetration by TALEN proteins. To test the feasibility of this approach, we conjugated *CCR5*-targeting TALEN proteins containing the high-activity Sharkey cleavage domain [35] with a thiol-reactive nitropyridyl (Npys) Arg_9_ (R9) peptide at various peptide-to-protein ratios [44], [45] (Fig. 2A and Fig. S2). Notably, the R9-CPP is commercially available and has been shown to effectively deliver several full-length proteins into mammalian cells [46], [47]. Because this linkage is reversible under reducing conditions, we anticipated that the R9-CPPs would be released from TALEN proteins by disulfide bond reduction following cytosolic entry. Each TALEN contained 18 Cys residues: one on each of the 17 TAL effector repeats and one on the surface of the FokI cleavage domain.

We observed that increasing the peptide-to-protein ratio led to incremental shifts in the molecular weight of TALEN proteins, as evidenced by native polyacrylamide gel electrophoresis (Fig. S3A). Direct monitoring of the 3-nitropyridine-2-thiol leaving group by reversed-phase HPLC further confirmed these findings (Fig. S3B). TALEN proteins conjugated to R9-CPP had a marked reduction in activity compared to non-conjugated protein (Fig. 2B). However, the efficiency of TALEN-mediated cleavage was restored by addition of 10 mM DTT (Fig. 2B and Fig. S2B), indicating that conjugation of the R9-CPP is reversible and does not dramatically inhibit TALEN cleavage efficiency under reducing conditions. Notably, we also found that TALEN protein labeled with R9-CPP at peptide-to-protein ratios greater than 30-to-1 had reduced activity even in the presence of DTT, suggesting that TALENs conjugated with excessive amounts of R9-CPPs may not have cleavage activity within a cell.

### TALEN Proteins Conjugated with CPPs are Cell-permeable, Induce Gene Knockout in Mammalian Cells, and are Non-toxic

**Figure 3:**
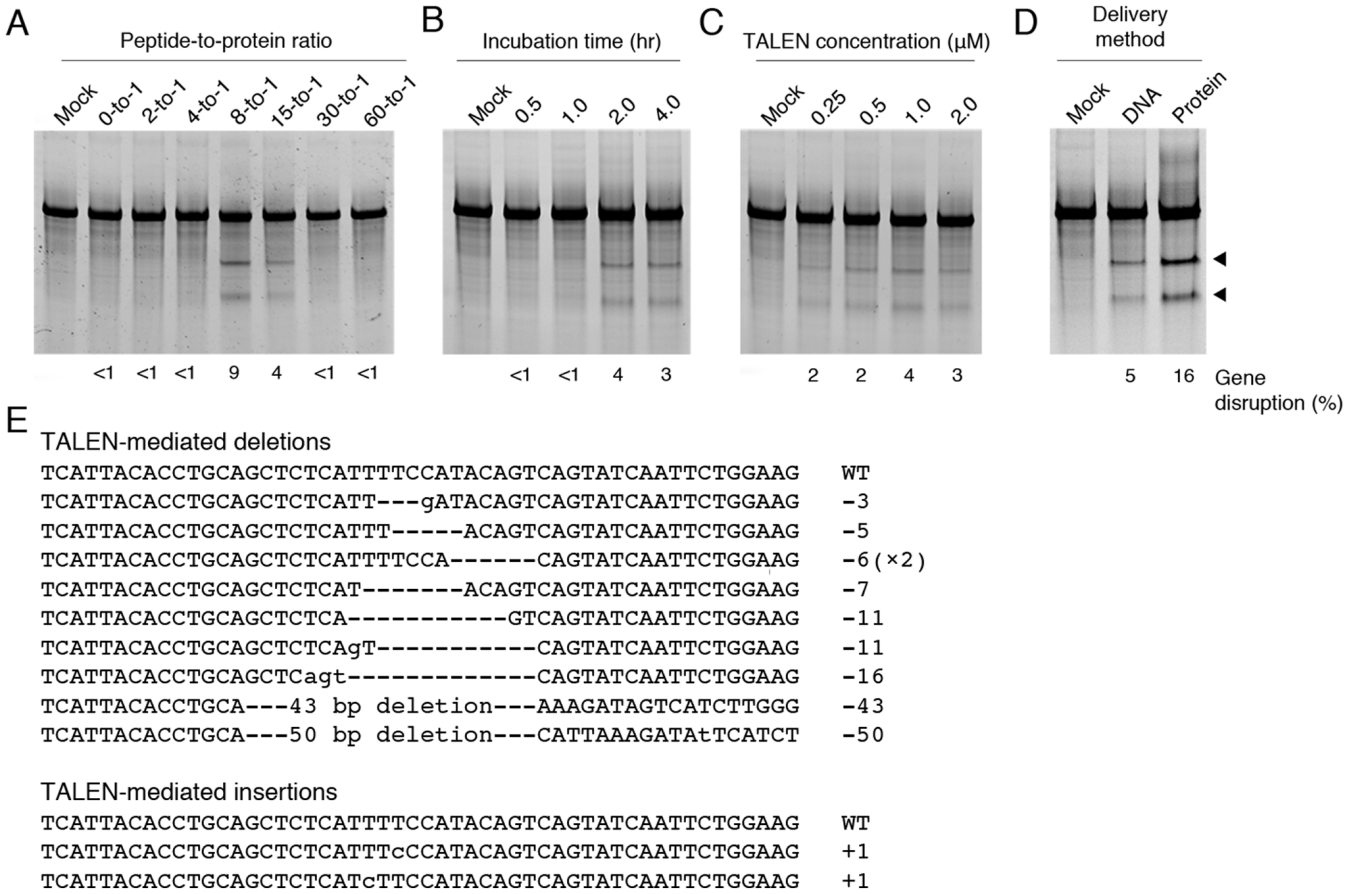
Modification of the endogenous *CCR5* gene by cell-permeable TALEN proteins. (A) Frequency of endogenous gene disruption in HeLa cells treated with *CCR5*-targeting TALEN proteins conjugated to R9-CPPs at various (A) peptide-to-protein ratios, (B) TALEN concentrations (µM), and (C) incubation periods (hr). (D) Comparison of gene disruption frequencies in HeLa cells transfected with 200 ng TALEN expression vectors or treated with 1.0 µM TALEN proteins for 2 hr. Gene mutagenesis frequencies were determined by the Surveyor nuclease assay. All R9-conjugated *CCR5*-targeted TALEN proteins were labeled in the presence of protease inhibitor cocktail. (E) Sequence analysis of the *CCR5* locus in HeLa cells treated with 1.0 µM conjugated TALEN proteins for 2 hr. Deletions (dashes) and insertions (lowercase) induced by NHEJ repair are aligned to the wild-type sequence (WT). Black triangles indicate expected Surveyor nuclease assay cleavage products.

To evaluate cell permeability of TALEN proteins conjugated with the R9-CPPs at various peptide-to-protein ratios, we measured R9-TALEN-mediated cleavage at the endogenous *CCR5* gene by the Surveyor nuclease assay [36]. We found that only TALEN proteins labeled with peptide-to-protein ratios of 8-to-1 and 15-to-1 were active (Fig. 3A). The lack of activity exhibited by TALENs labeled at low peptide-to-protein ratios is most likely a product of too few R9 groups present on the surface of the TALEN to confer sufficient cell-penetrating functionality. As suggested by data from the *in vitro* cleavage assay, the low activity observed for TALENs labeled at high peptide-to-protein ratios is most likely due to incomplete reduction following cytosolic entry.

We next evaluated whether incubation time or TALEN concentration affected cell-permeability and gene disruption. We previously showed that both parameters are important factors for maximizing the activity of cell-permeable ZFN proteins [29]. We found that 2-hr incubation periods with 1 µM TALEN protein provided the highest rates of gene disruption (Fig. 3B and 3C). Furthermore, inclusion of protease inhibitor cocktail (PIC) in the R9-conjugation buffer enhanced the efficiency of TALEN-mediated gene knockout (Fig. S4). HeLa cells treated with R9-conjugated TALEN proteins under these conditions showed *CCR5* gene disruption frequencies of ∼16%, nearly 3-fold higher than the levels of gene disruption achieved by transient transfection of TALEN expression vectors (Fig. 3D). Sequence analysis of cloned *CCR5* alleles amplified from treated HeLa cells confirmed the presence of TALEN-induced insertions and deletions in the *CCR5* gene (Fig. 3E). Notably, unlike cell-permeable ZFN proteins, we found that the efficiency of R9-conjugated TALEN-mediated gene knockout did not increase with consecutive protein treatments, presumably due to the inhibitory effects of excess unconjugated R9-CPP. Together, these studies indicate that TALEN proteins modified with R9-CPPs are cell-permeable and disrupt the endogenous *CCR5* gene with high efficiency.

**Figure 4:**
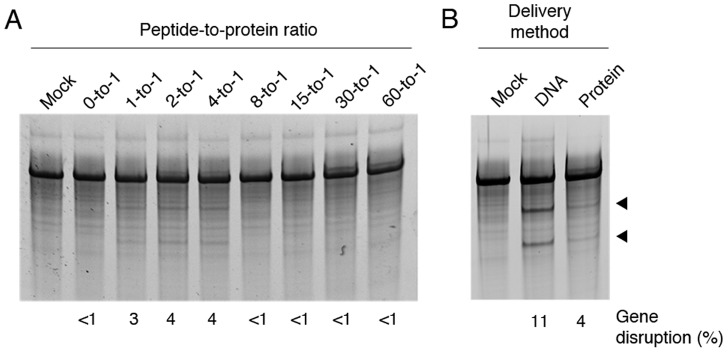
Modification of the endogenous *BMPR1A* gene by cell-permeable TALEN proteins. (A) Frequency of gene disruption in HEK293 cells treated for 2 hr with 1.0 µM *BMPRIA*-targeting TALEN proteins conjugated at various peptide-to-protein ratios. All R9-conjugated *BMPR1A*-targeted TALEN proteins were labeled in the presence of protease inhibitor cocktail. (B) Comparison of the frequency of *BMPR1A* knockout in HEK293 cells transfected with 200 ng of TALEN expression vectors or treated with 1.0 µM cell-permeable TALEN proteins for 2 hr. Black triangles indicate expected Surveyor nuclease assay cleavage products.

To test the general utility of this approach, we treated human embryonic kidney (HEK) 293 cells with Sharkey-containing TALEN proteins designed to target the human bone morphogenetic protein receptor type IA (*BMPR1A*) gene [34]. We observed that the R9-conjugated *BMPR1A*-targeting TALENs labeled in the presence of PIC were capable of inducing gene knockout at several peptide-to-protein ratios (Fig. 4A and B). However, these proteins displayed lower activity than those targeting the *CCR5* gene. Surprisingly, we found that no gene disruption was evident at peptide-to-protein ratios shown to be effective for the *CCR5*-targeting TALENs, suggesting that different TALEN proteins may display distinct conjugation characteristics. Sequence analysis of cloned alleles confirmed targeted modification of the *BMPR1A* gene in HEK293 cells.

We observed no apparent reduction in cell viability in either HeLa or HEK293 cells after treatment with various concentrations of R9-conjugated TALENs (Fig. S5). We have, however, observed increased toxicity in relation to decreased TALEN protein purity, suggesting that TALEN purification is an important factor to consider for protein-based genome editing applications in sensitive cell types.

## Discussion

We show here that poly-Arg (R9) cell-penetrating peptides (CPPs) can be reversibly conjugated to Cys residues present on the surface of TAL effector repeat domains to impart cell-penetrating activity onto TALEN proteins. R9-conjugated TALENs induced gene knockout of the human *CCR5* and *BMPR1A* genes in HeLa and HEK293 cells, respectively. TALEN protein delivery resulted in similar levels of gene knockout as transient transfection of TALEN expression vectors, and the R9-conjugated TALEN proteins did not affect cell viability. Ru *et al* recently reported that TAT-TALEN fusion proteins are cell-permeable and capable of inducing knockout of the *CCR5* gene at frequencies ∼16% in HeLa cells and ∼3% in induced pluripotent stem cells [31]. Although these results are promising, this approach could be technically limited, as TAT fusion proteins are often difficult to express and purify. For example, we have thus far been unable to produce sufficient quantities of TAT-TALEN or TAT-ZFN fusion proteins for analysis in cell culture. Here, we conjugated cell-penetrating activity to the surface of the purified TALEN protein. Because unmodified TALEN proteins can be purified to high yields, this approach is scalable. Our conjugation method is relatively efficient: We observed up to 50% conjugation of TALEN proteins at 15-to-1 peptide-to-protein ratios. We note that this conjugation strategy can be used to attach various chemical functionalities onto the surface of the TALEN protein. By conjugating cell-surface receptor ligands to the TALEN protein, cell-type specific delivery of TALEN proteins may be possible.

We found that unreacted R9-CPPs were not easily removed from the conjugation reactions using commercial spin concentrators or size-exclusion columns, indicating that stepwise buffer exchange may be necessary for purification. Although TALEN proteins applied to cells immediately after conjugation demonstrated negligible toxicity, we suspect that further purification to remove unreacted CPP might improve TALEN activity. Unreacted CPPs might also compete with conjugated TALENs for association with negatively charged membrane components that coordinate cellular internalization. Advances in the removal of unconjugated CPPs might therefore enable higher levels of gene knockout by allowing for consecutive protein treatments.

We found that peptide-to-protein ratio in the TALEN conjugation reaction was important for gene knockout efficiency. We suspect this is due to a balance between the number of CPPs required to impart cell-internalizing activity onto the TALEN protein and the extent to which these peptides are removed via reduction following cytosolic entry. Our data indicate that TALEN proteins over-saturated with the R9-CPP have the greatest degree of conjugation but also the lowest degree of gene silencing activity. We also found that TALEN protein concentration and incubation time with cells were two crucial parameters for maximizing gene knockout activity. After optimization, we observed that a single TALEN protein treatment led to knockout frequencies of greater than 15% in HeLa cells. This mutagenesis frequency is similar to that reported for HeLa cells treated consecutively with purified TAT-TALEN proteins [31], indicating that R9-conjugated TALENs may be internalized more efficiently. Surprisingly, R9-conjugated TALENs targeting the *BMPR1A* gene showed lower levels of mutagenesis than the *CCR5*-targeting TALENs. The exact reason for this discrepancy remains unknown; however, we suspect that the DNA binding affinity of individual TALEN monomers play a critical role in activity. Further experiments are required to determine whether conjugation with other types of CPPs further enhance the cell penetration of TALENs. Additionally, we did not examine whether R9-conjugated TALEN proteins mediate gene knockout in post-mitotic cells. As such, future studies will be aimed at determining the efficiency of protein-based gene knockout methods in non-dividing cells. Finally, given the potential of this chemical approach to protein transduction, this approach might also be an effective means for delivering purified CRISPR/Cas9 components directly into cells [8], [48].

## Supporting Information

Figure S1 **Genetically fused cell-penetrating peptides (CPPs) fail to deliver TALEN proteins into mammalian cells.** (A) (Top) Amino acid sequences of the CPPs used in this study. (Bottom) SDS-PAGE of purified CPP-TALEN fusion proteins. (B) *In vitro* cleavage activities of purified CPP-TALEN fusion proteins. (C and D) Frequency of endogenous *CCR5* gene disruption in (C) HeLa cells treated with 2.0 µM purified CPP-TALEN fusion proteins for 2 hr and (D) HEK293 reporter cells transfected with expression vectors encoding CPP-TALENs. Error bars indicate standard deviation (*n* = 3). (E) Western blot of lysate from HeLa cells treated with 2.0 µM purified CPP-TALEN fusion proteins for 2 hr. Samples were probed with horseradish peroxidase-conjugated anti-FLAG antibody. The internal loading control was β-actin, detected with peroxidase-conjugated anti-β-actin antibody. Purified R9-conjugated TALEN proteins were used as a positive control.
(TIF)

Figure S2 **Characterization of purified TALEN proteins.** **(A)** SDS-PAGE of purified left (L) and right (R) *CCR5*-targeting TALEN proteins. (B) *In vitro* cleavage assay of purified *CCR5*-targeting TALEN proteins.
(TIF)

Figure S3 **The extent of CPP-conjugation to TALEN protein is dependent on the peptide-to-protein ratio.** (A) Native PAGE of 2.0 µM TALEN protein reacted with the R9-CPP at various peptide-to-protein ratios. Reactions were performed at room temperature for 4 hr. (B) C_18_ reversed-phase HPLC traces of the TALEN and R9-CPP conjugation reaction at t = 0 hr (red) and t = 1 hr (blue). Reaction progress was determined by monitoring 3-nitropyridine-2-thiol formation at 350 nm. The retention time of the leaving group matched that of the commercially available compound. Generation of a standard curve indicated that the conjugation efficiency was ∼50% at 1 hr.
(TIF)

Figure S4 **Protease inhibitor cocktail (PIC) is required for TALEN delivery into mammalian cells.** Frequency of endogenous *CCR5* gene disruption in HeLa cells treated with 2.0 µM purified R9-labeled TALEN proteins for 4 hr in the presence or absence of PIC as determined by the Surveyor nuclease assay. Experiments were performed in duplicate (mock) or triplicate (plus or minus PIC).
(TIF)

Figure S5 **Toxicity of R9-labeled TALEN proteins.** Viability of HeLa and HEK293 cells treated with various concentrations of R9-conjugated *CCR5*-targeting TALEN proteins for 2 hr. Error bars indicate standard deviation (*n* = 3).
(TIF)

Table S1 **Amino acid sequences of the TALENs used in this study.** N- and C-terminal TALE domains are colored green, TALE repeats are colored black and the Sharkey cleavage domain is colored purple. RVDs are highlighted red.
(DOCX)

Table S2 **Primers used to amplify the endogenous** ***CCR5*** **and** ***BMPR1A*** **genes.**
(DOCX)
