# Supplementary figures and images for: Cell-Penetrating Peptide-Mediated Delivery of TALEN Proteins via Bioconjugation for Genome Engineering
Source: PLoS One. 2014 Jan 20;9(1):e85755. doi: 10.1371/journal.pone.0085755 (PMC3896395; doi:10.1371/journal.pone.0085755)

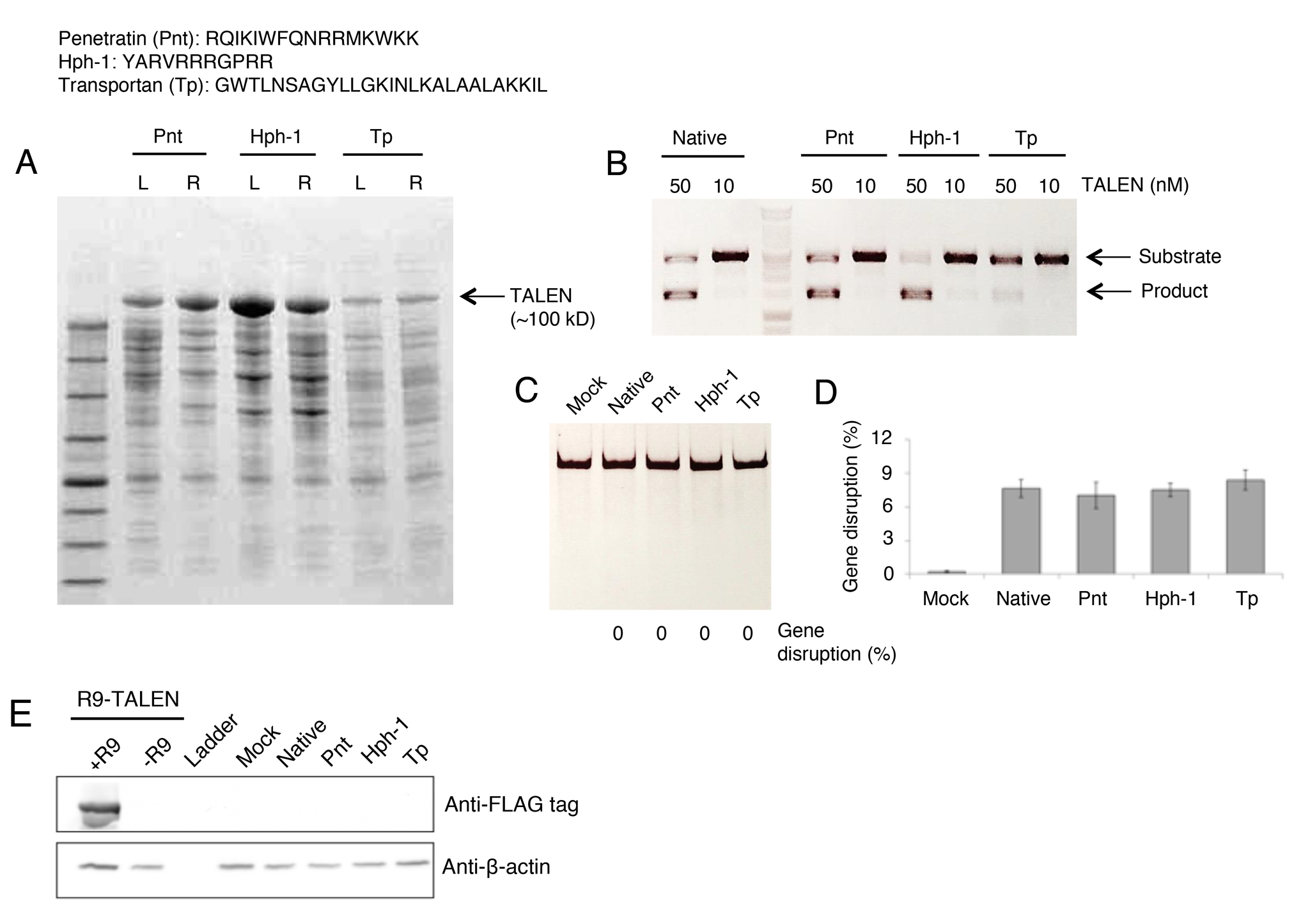

Supplement: Figure S1 — Genetically fused cell-penetrating peptides (CPPs) fail to deliver TALEN proteins into mammalian cells. (A) (Top) Amino acid sequences of the CPPs used in this study. (Bottom) SDS-PAGE of purified CPP-TALEN fusion proteins. (B) In vitro cleavage activities of purified CPP-TALEN fusion proteins. (C and D) Frequency of endogenous CCR5 gene disruption in (C) HeLa cells treated with 2.0 µM purified CPP-TALEN fusion proteins for 2 hr and (D) HEK293 reporter cells transfected with expression vectors encoding CPP-TALENs. Error bars indicate standard deviation (n = 3). (E) Western blot of lysate from HeLa cells treated with 2.0 µM purified CPP-TALEN fusion proteins for 2 hr. Samples were probed with horseradish peroxidase-conjugated anti-FLAG antibody. The internal loading control was β-actin, detected with peroxidase-conjugated anti-β-actin antibody. Purified R9-conjugated TALEN proteins were used as a positive control. (TIF) [file pone.0085755.s001.tif]

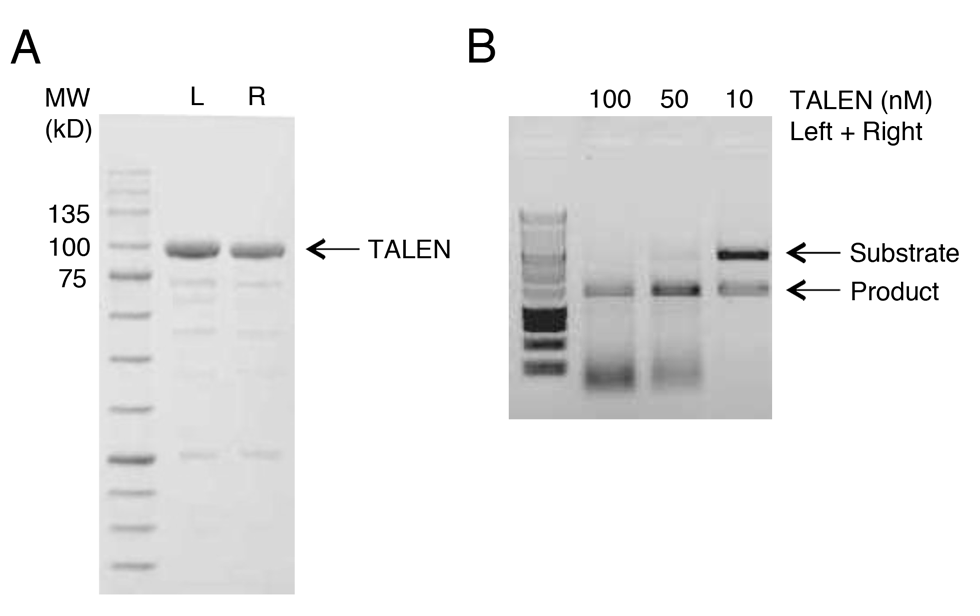

Supplement: Figure S2 — Characterization of purified TALEN proteins. (A) SDS-PAGE of purified left (L) and right (R) CCR5-targeting TALEN proteins. (B) In vitro cleavage assay of purified CCR5-targeting TALEN proteins. (TIF) [file pone.0085755.s002.tif]

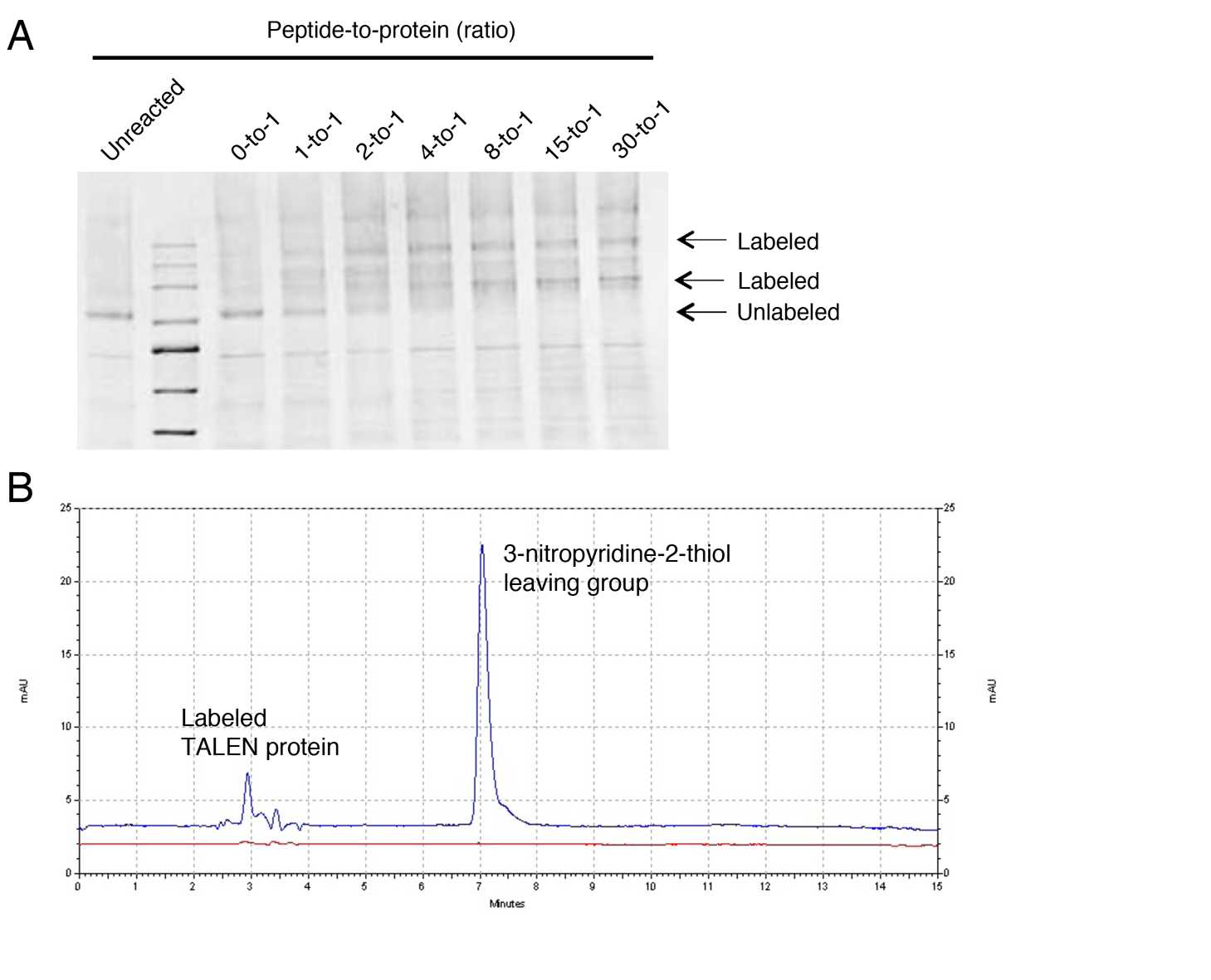

Supplement: Figure S3 — The extent of CPP-conjugation to TALEN protein is dependent on the peptide-to-protein ratio. (A) Native PAGE of 2.0 µM TALEN protein reacted with the R9-CPP at various peptide-to-protein ratios. Reactions were performed at room temperature for 4 hr. (B) C18 reversed-phase HPLC traces of the TALEN and R9-CPP conjugation reaction at t = 0 hr (red) and t = 1 hr (blue). Reaction progress was determined by monitoring 3-nitropyridine-2-thiol formation at 350 nm. The retention time of the leaving group matched that of the commercially available compound. Generation of a standard curve indicated that the conjugation efficiency was ∼50% at 1 hr. (TIF) [file pone.0085755.s003.tif]

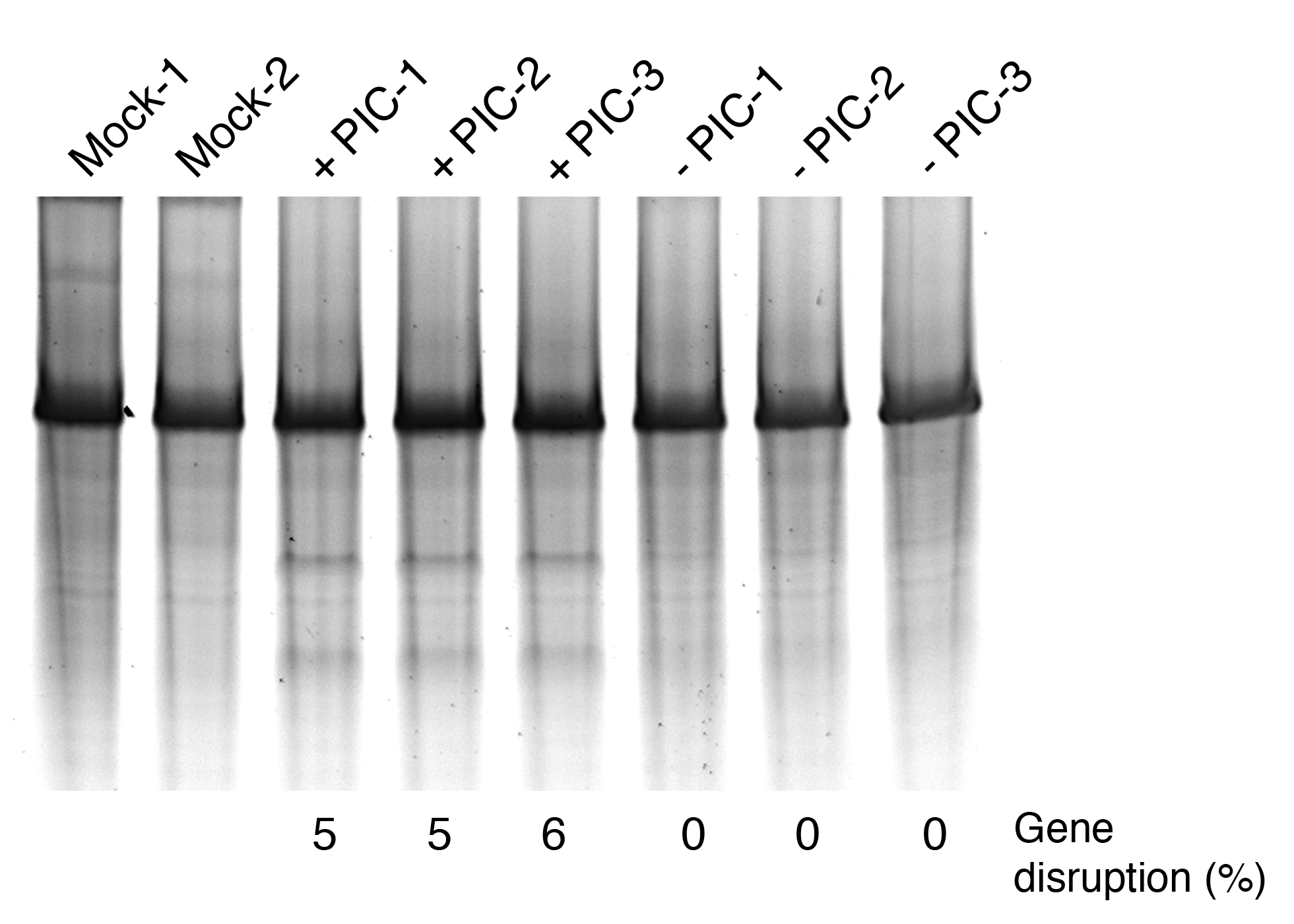

Supplement: Figure S4 — Protease inhibitor cocktail (PIC) is required for TALEN delivery into mammalian cells. Frequency of endogenous CCR5 gene disruption in HeLa cells treated with 2.0 µM purified R9-labeled TALEN proteins for 4 hr in the presence or absence of PIC as determined by the Surveyor nuclease assay. Experiments were performed in duplicate (mock) or triplicate (plus or minus PIC). (TIF) [file pone.0085755.s004.tif]

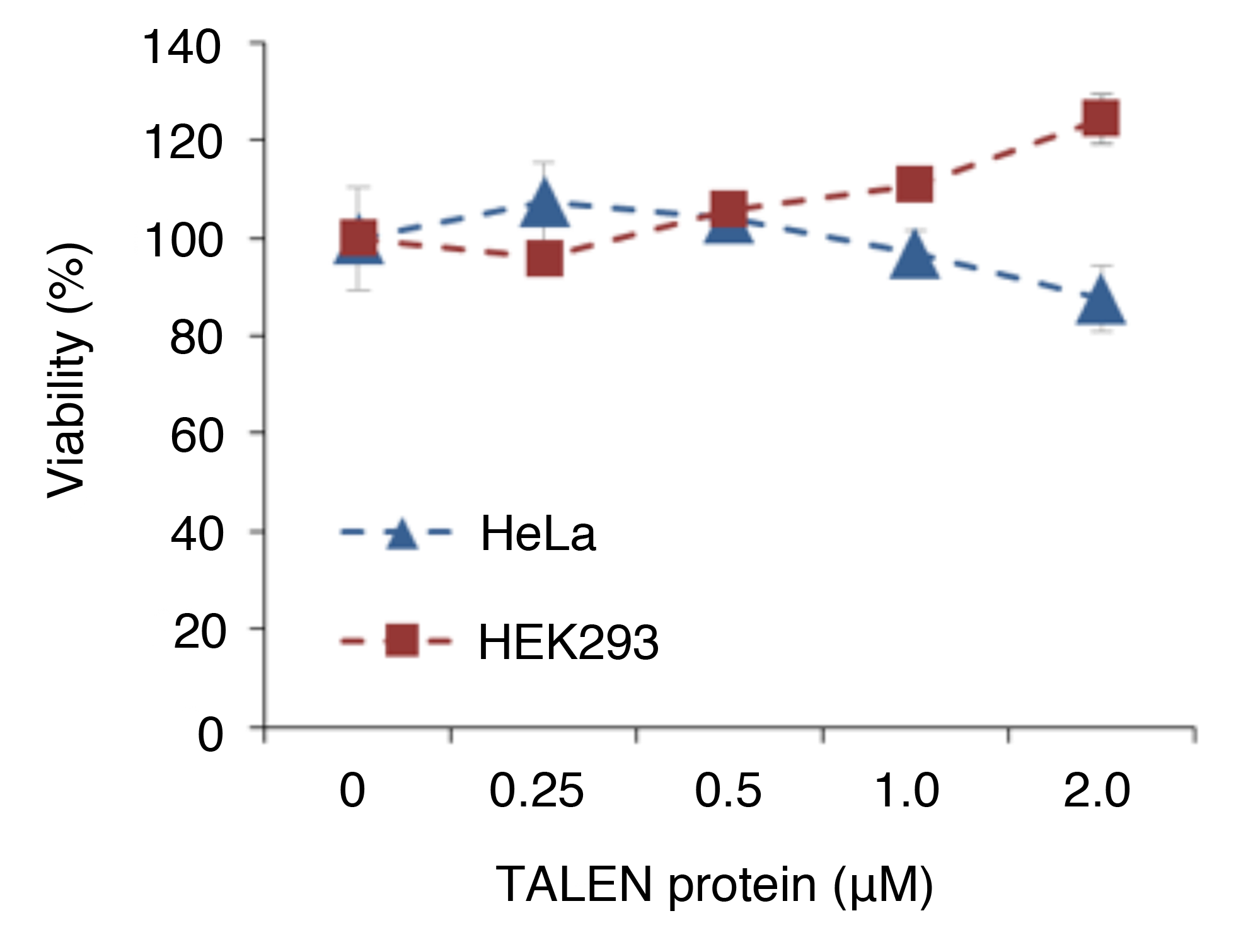

Supplement: Figure S5 — Toxicity of R9-labeled TALEN proteins. Viability of HeLa and HEK293 cells treated with various concentrations of R9-conjugated CCR5-targeting TALEN proteins for 2 hr. Error bars indicate standard deviation (n = 3). (TIF) [file pone.0085755.s005.tif]
