# Supplementary material for: Cell-Penetrating Peptide-Mediated Delivery of TALEN Proteins via Bioconjugation for Genome Engineering
Source: PLoS One. 2014 Jan 20;9(1):e85755. doi: 10.1371/journal.pone.0085755 (PMC3896395; doi:10.1371/journal.pone.0085755)
Supplement: Table S1 — Amino acid sequences of the TALENs used in this study. N- and C-terminal TALE domains are colored green, TALE repeats are colored black and the Sharkey cleavage domain is colored purple. RVDs are highlighted red. (DOCX) [file pone.0085755.s006.docx]

>Left CCR5 TALEN

MAHHHHHHASMDYKDHDGDYKDHDIDYKDDDDKMAPKKKRKVGIHRGVPMVDLRTLGYSQQQQEKIKPKVRSTVAQHHEALVGHGFTHAHIVALSQHPAALGTVAVKYQDMIAALPEATHEAIVGVGKQWSGARALEALLTVAGELRGPPLQLDTGQLLKIAKRGGVTAVEAVHAWRNALTGAPLNLTPDQVVAIASNGGGKQALETVQRLLPVLCQDHGLTPEQVVAIASHDGGKQALETVQRLLPVLCQAHGLTPDQVVAIASNIGGKQALETVQRLLPVLCQAHGLTPAQVVAIASNGGGKQALETVQRLLPVLCQDHGLTPDQVVAIASNGGGKQALETVQRLLPVLCQDHGLTPEQVVAIASNIGGKQALETVQRLLPVLCQAHGLTPDQVVAIASHDGGKQALETVQRLLPVLCQAHGLTPAQVVAIASNIGGKQALETVQRLLPVLCQDHGLTPDQVVAIASHDGGKQALETVQRLLPVLCQDHGLTPEQVVAIASHDGGKQALETVQRLLPVLCQAHGLTPDQVVAIASNGGGKQALETVQRLLPVLCQAHGLTPAQVVAIANNNGGKQALETVQRLLPVLCQDHGLTPDQVVAIASHDGGKQALETVQRLLPVLCQDHGLTPEQVVAIASNIGGKQALETVQRLLPVLCQAHGLTPDQVVAIANNNGGKQALETVQRLLPVLCQAHGLTPAQVVAIASHDGGKQALETVQRLLPVLCQDHGLTPEQVVAIASNGGGRPALESIVAQLSRPDPALAALTNDHLVALACLGGRPALDAVKKGLPHAPALIKRTNRRIPERTSHRVAGSQLVKSELEEKKSELRHKLKYVPHEYIELIEIARNPTQDRILEMKVMEFFMKVYGYRGEHLGGSRKPDGAIYTVGSPIDYGVIVDTKAYSGGYNLPIGQADEMQRYVEENQTRNKHINPNEWWKVYPSSVTEFKFLFVSGHFKGNYKAQLTRLNHITNCNGAVLSVEELLIGGEMIKAGTLTLEEVRRKFNNGEINF

>Right CCR5 TALEN RVDs

LTPDQVVAIASHDGGKQALETVQRLLPVLCQDHG

LTPEQVVAIASNGGGKQALETVQRLLPVLCQAHG

LTPDQVVAIASNGGGKQALETVQRLLPVLCQAHG

LTPAQVVAIASHDGGKQALETVQRLLPVLCQDHG

LTPDQVVAIASHDGGKQALETVQRLLPVLCQDHG

LTPEQVVAIASNIGGKQALETVQRLLPVLCQAHG

LTPDQVVAIANNNGGKQALETVQRLLPVLCQAHG

LTPAQVVAIASNIGGKQALETVQRLLPVLCQDHG

LTPDQVVAIASNIGGKQALETVQRLLPVLCQDHG

LTPEQVVAIASNGGGKQALETVQRLLPVLCQAHG

LTPDQVVAIASNGGGKQALETVQRLLPVLCQAHG

LTPAQVVAIANNNGGKQALETVQRLLPVLCQDHG

LTPDQVVAIASNIGGKQALETVQRLLPVLCQDHG

LTPEQVVAIASNGGGKQALETVQRLLPVLCQAHG

LTPDQVVAIASNIGGKQALETVQRLLPVLCQAHG

LTPAQVVAIASHDGGKQALETVQRLLPVLCQDHG

LTPEQVVAIASNGGGRPALE

>Left BMPR1A TALEN

LTPDQVVAIASNIGGKQALETVQRLLPVLCQDHG

LTPEQVVAIASHDGGKQALETVQRLLPVLCQAHG

LTPDQVVAIASNIGGKQALETVQRLLPVLCQAHG

LTPAQVVAIASNIGGKQALETVQRLLPVLCQDHG

LTPDQVVAIASNGGGKQALETVQRLLPVLCQDHG

LTPEQVVAIASNGGGKQALETVQRLLPVLCQAHG

LTPDQVVAIANNNGGKQALETVQRLLPVLCQAHG

LTPAQVVAIASNIGGKQALETVQRLLPVLCQDHG

LTPDQVVAIASNIGGKQALETVQRLLPVLCQDHG

LTPEQVVAIASHDGGKQALETVQRLLPVLCQAHG

LTPDQVVAIASNIGGKQALETVQRLLPVLCQAHG

LTPAQVVAIANNIGGKQALETVQRLLPVLCQDHG

LTPDQVVAIASNGGGKQALETVQRLLPVLCQDHG

LTPEQVVAIANNNGGKQALETVQRLLPVLCQAHG

LTPDQVVAIANHDGGKQALETVQRLLPVLCQAHG

LTPAQVVAIASHDGGKQALETVQRLLPVLCQDHG

LTPEQVVAIASNGGGRPALE

>Left BMPR1A TALEN

LTPDQVVAIASNIGGKQALETVQRLLPVLCQDHG

LTPEQVVAIANNNGGKQALETVQRLLPVLCQAHG

LTPDQVVAIANNNGGKQALETVQRLLPVLCQAHG

LTPAQVVAIASHDGGKQALETVQRLLPVLCQDHG

LTPDQVVAIASNGGGKQALETVQRLLPVLCQDHG

LTPEQVVAIASHDGGKQALETVQRLLPVLCQAHG

LTPDQVVAIANHDGGKQALETVQRLLPVLCQAHG

LTPAQVVAIASHDGGKQALETVQRLLPVLCQDHG

LTPDQVVAIASNIGGKQALETVQRLLPVLCQDHG

LTPEQVVAIASNIGGKQALETVQRLLPVLCQAHG

LTPDQVVAIASNGGGKQALETVQRLLPVLCQAHG

LTPAQVVAIANNIGGKQALETVQRLLPVLCQDHG

LTPDQVVAIASNIGGKQALETVQRLLPVLCQDHG

LTPEQVVAIASNGGGKQALETVQRLLPVLCQAHG

LTPDQVVAIASHDGGKQALETVQRLLPVLCQAHG

LTPAQVVAIASNGGGKQALETVQRLLPVLCQDHG

LTPEQVVAIANNNGGRPALE

**Table S1. Amino acid sequences of the TALENs used in this study.** N- and C-terminal TALE domains are colored green, TALE repeats are colored black and the Sharkey cleavage domain is colored purple. RVDs are highlighted red.
