# Supplementary material for: Cell-Penetrating Peptide-Mediated Delivery of TALEN Proteins via Bioconjugation for Genome Engineering
Source: PLoS One. 2014 Jan 20;9(1):e85755. doi: 10.1371/journal.pone.0085755 (PMC3896395; doi:10.1371/journal.pone.0085755)
Supplement: Table S2 — Primers used to amplify the endogenous CCR5 and BMPR1A genes. (DOCX) [file pone.0085755.s007.docx]

| Primer name | Primer sequence (5’-to-3’) |
| --- | --- |
| CCR5 external 5' | ACAGTTTGCATTCATGGAGGGC |
| CCR5 external 3' | AACTGAGCTTGCTCGCTCGG |
| CCR5 internal 5' (BamHI site underlined) | CGCGGATCCTTAAAAGCCAGGACGGTCAC |
| CCR5 internal 3' (EcoRI site underlined) | CCGGAATTCTGTAGGGAGCCCAGAAGAGA |
| BMPR1A external 5' | AATTAGAACAGCCAGAAAGG |
| BMPR1A external 3' | CCTGGCCAATAAAATGATTT |
| BMPR1A internal 5' (BamHI site underlined) | CGCGGATCCGGTGTGTTTGGGCATTTGTTTCC |
| BMPR1A internal 3' (EcoRI site underlined) | CCGGAATTCGGAATTTTGAAGCTCAGCCTGGA |

**Table S2. Primers used to amplify the endogenous *CCR5* and *BMPR1A* genes.**
